# Endoplasmic Reticulum Stress-Mediated Hippocampal Neuron Apoptosis Involved in Diabetic Cognitive Impairment

**DOI:** 10.1155/2013/924327

**Published:** 2013-04-24

**Authors:** Xiaoming Zhang, Linhao Xu, Daqiang He, Shucai Ling

**Affiliations:** Department of Anatomy and Cell Biology, School of Medicine, Zhejiang University, 866 Yuhangtang Road, Hangzhou 310058, China

## Abstract

Poor management of DM causes cognitive impairment while the mechanism is still unconfirmed. The aim of the present study was to investigate the activation of C/EBP Homology Protein (CHOP), the prominent mediator of the endoplasmic reticulum (ER) stress-induced apoptosis under hyperglycemia. We employed streptozotocin- (STZ-) induced diabetic rats to explore the ability of learning and memory by the Morris water maze test. The ultrastructure of hippocampus in diabetic rats and cultured neurons in high glucose medium were observed by transmission electron microscopy and scanning electron microscopy. TUNEL staining was also performed to assess apoptotic cells while the expression of CHOP was assayed by immunohistochemistry and Western blot assay in these hippocampal neurons. Six weeks after diabetes induction, the escape latency increased and the average frequency in finding the platform decreased in diabetic rats (*P* < 0.05). The morphology of neuron and synaptic structure was impaired; the number of TUNEL-positive cells and the expression of CHOP in hippocampus of diabetic rats and high glucose medium cultured neurons were markedly altered (*P* < 0.05). The present results suggested that the CHOP-dependent endoplasmic reticulum (ER) stress-mediated apoptosis may be involved in hyperglycemia-induced hippocampal synapses and neurons impairment and promote the diabetic cognitive impairment.

## 1. Introduction

Diabetes mellitus (DM) is a chronic metabolic disease and its prevalence is increasing worldwide. Poor management of DM causes several systemic complications, such as diabetic retinopathy, diabetic nephropathy, and diabetic neuropathy [1–3] through multiple mechanisms, including nonenzymatic glycation, protein kinase C activation, increased aldose reductase activity, and oxidative stress that contributes to the pathogenesis of the disease and the development of complications as well [4–9]. Diabetes is also associated with cognitive defect, including impaired performance in cognition and memory [10, 11]. The cognitive decrements may be connected to diabetes-related comorbidities [12]. It has been reported that cognitive dysfunction is present in 30% to 40% of elderly DM patients, and the severity of cognitive impairment has a direct relationship with poor glycemic control in these patients [13]. DM patients show an increase in the risk of dementia in general [11], vascular dementia in particular [14], and poststroke dementia [15]. The study shows that diabetes affects the content of several exocytotic proteins in hippocampus and induced cognitive impairment and memory loss in diabetic humans and animal models [16]. Our previous studies also revealed that diabetes gave rise to cognitive impairment and significantly decreased the synapse in the hippocampus of diabetic rats [17], while the mechanism of hyperglycemia-induced hippocampal neuron impairment is still elusive. 

The endoplasmic reticulum (ER) is an organelle, which is susceptible to various type of injury, causes inhibition of protein synthesis, and delivery of apoptotic signals. The destructive stimulus may impair the ER function and leads to the activation of unfolded-protein-response-ER stress [18, 19]. Under hyperglycemic conditions, the endothelial cell was dysfunction and increased the oxidative stress and concomitantly activated the ER stress [20]. When stress is present, the expression of chaperones would be activated, protein translation would be attenuated, and ER-associated degradation would be activated. Eventually, the cell might be subject to apoptosis under prolonged ER stress environment [21]. C/EBP Homology Protein (CHOP) is a prominent protein involved in the ER stress-mediated cell apoptosis [22, 23]. CHOP-mediated cell death promoted several neurodegenerative diseases [24, 25] and impairment of memory and cognitive functions [26]. 

ER stress-induced apoptosis was increasingly acknowledged as an important mechanism in the development of diabetes [27]; therefore, we hypothesized that endoplasmic reticulum stress mediator, CHOP-induced hippocampal neuron apoptosis, may play a role in the diabetic cognitive impairment. 

## 2. Materials and Methods

### 2.1. Animal Model Induction

Thirty Sprague-Dawley (SD) rats (from Experiment Animal Center of Zhejiang University) were randomly divided into two groups, the diabetic group and the control group. Fifteen rats were fasted for 10 h before streptozotocin (STZ) (Alexis Corporation, Switzerland) was injected into the caudal vein (30 mg/kg) to induce diabetes; the remaining 15 rats were also fasted for 10 h and then received an injection of 0.9% saline as the control group. The plasma glucose level was measured by a Glucose Electrode Calibrator (MediSense QA2583-3364, USA), and the urine glucose level was measured by test strips for urine glucose (Anjian GZZJ 1-2002, China). Forty-eight hours after the injection of STZ, the blood glucose >16 mmol/L and the urine glucose > (+) were regarded as successful induction of diabetes. All procedures were carried out under the National Institutes of Health guide for the care and use of laboratory animals. Animal ethical permission for this study was granted by the Ethical Committee of the School of Medicine, Zhejiang University. Furthermore, all efforts were made to minimize the rats' suffering during the experiment.

### 2.2. Cell Culture

Two adult male and three female SD rats were closed in the same cage just one day. On the next day, we will separate the male and female rats, and we start counting from this day. On 18th days the pregnant rats are used for doing experiment. Hippocampal neurons were isolated from embryonic day 18 of SD rats. The tissues of the hippocampus were cut into small pieces, digested with trypsin, dissociated with a fire polished glass pipette, and centrifuged to separate undissociated tissue. Cells were then seeded into 24-well cell culture plates in serum-free Neurobasal medium supplemented with 2% B_27_ and 0.05 mM glutamine to observe survival and neurite outgrowth. All culture reagents were purchased from GIBCO (Grand Island, USA). The hippocampal neurons were cultured till the 6th day, then the half neurons were cultured with high glucose Neurobasal medium (contained 100 mM glucose) for 24 h as the high glucose group, and the rest of the neurons were cultured with the normal Neurobasal medium (contained basal 25 mM glucose) as the control group. For six-well plates, it is about 500,000 neurons in each well which is used for Western blot detection. For 48-well plates, it is approximately 3,000–4,000 in each well for staining exam. 

### 2.3. Morris Water Maze Test

Six weeks after diabetes induction, the spatial learning performance in both groups was tested by the Morris water maze (electric factory of Anhui, China). A circular, black painted pool (150 cm in diameter, 50 cm in length, and 30 cm in depth) was filled with opaque water by the addition of 30 mL of black ink. An invisible platform (8 cm diameter) was submerged 1 cm below the water line and placed in the center of the northeast quadrant which was determined with four starting locations labelled north (N), east (E), south (S), and west (W) at equal distance on the rim. During three consecutive days at 7 a.m. in the morning, the rats were trained four times per day, given a maximum of 120 s to escape onto the hidden platform and were allowed to stay on it for 30 s. Rats who failed to locate the platform would be placed on it. Each rat was gently placed into the water with the nose facing the wall at one of the starting points. All the animals were tested in sequence and the time intervals between two trials was about 1–1.5 h. The animals were put in the same starting points on each day. The escape latency was averaged from four trials per day. The platform was removed from the pool on the 4th day and the average frequency in finding the location of platform in all rats will be recorded in 120 s. 

### 2.4. Transmission Electron Microscopy and Scanning Electron Microscopy Observation

After the Morris water maze test, five rats per group were anaesthetized by a lethal intraperitoneal injection of Nembutal and the rat's hippocampal tissues were processed for the transmission electron microscope (TEM) observation. The tissues were separated of five rats per group, then rinsed in cold phosphate-buffered saline (PBS) and placed in 2.5% glutaraldehyde at 4°C for 4 h. The tissues were rinsed in buffer and postfixed with 1% osmium tetroxide for 1 h, undergoing a graded ethanol dehydration series, and infiltrated using a mixture of one-half propylene oxide and resin overnight. Twenty-four hours later, the tissues were embedded in resin. The 120 nm sections were cut and stained with 4% uranyl acetate for 20 min and with 0.5% lead citrate for 5 min. The ultrastructure of the hippocampus was observed under the transmission electron microscopy (Philips Tecnai 10, Holland).

The cultured neurons were for the scanning electron microscopy (SEM) observation. The hippocampal neurons of two groups were cultured on poly-L-lysine-coated glass cover slides, fixed with 1% glutaraldehyde (pH7.4), postfixed with 1% osmium tetroxide, dehydrated through an ascending series of ethanol and isoamyl acetate, and dried through a CO_2_ critical point method. The neurons were ion-coated with a thin layer of gold and were examined by the Hitachi scanning electron microscope (Cambridge, S-260, UK).

### 2.5. TUNEL Staining, Immunohistochemistry Assay

After the Morris water maze test, five rats per group were anaesthetized by a lethal intraperitoneal injection of Nembutal. The animals were perfused with 100 mL of normal saline and 250 mL of 4% formaldehyde by the aorta cannulation for 20–30 min. The brain of each rat was embedded in paraffin, and transverse paraffin sections (5 *μ*m thick) were mounted on silane-coated slides for TUNEL and immunohistochemistry staining. 

TUNEL staining was performed to assess apoptotic cells in hippocampus of diabetic rats according to the manufacturer's protocol. The cells labelled with brown were counted under the Nikon E200 microscope (Nikon Corporation, Japan). 

For immunohistochemistry, the sections of tissues and the slides of cultured neurons were washed in 0.01 M PBS containing 0.3% Triton X-100 (pH 7.4, PBS-T), then immersed in 2% normal horse serum in PBS for 120 min at 37°C, incubated overnight at 4°C with polyclone CHOP antibody (1 : 100, Santa Cruz Biotechnology, USA) in PBS containing 1% bovine serum albumin, washed with PBS (3 × 5 min), incubated in biotinylated horse-anti-mouse IgG (1 : 200, Boster Biotechnology, China) in PBS for 2 h at room temperature, washed with PBS-T (3 × 5 min), then incubated in avidin-biotin-peroxidase complex solution (ABC, 1 : 100, Boster Biotechnology, China) for 2 h at room temperature, and then rinsed again with PBS-T (3 × 5 min). Visualization was made by incubating the tissue for 10 min in 0.04% 3-diaminobenzidine containing 0.01% H_2_O_2_. Rat immunoglobulin IgG (1 : 200, Biomeda Corporation, USA) was used instead of primary antibody as a negative control. 

### 2.6. Western Blot Assay

The hippocampus of the remaining five rats in each group and the rest of the cultured neurons were snap-frozen in liquid nitrogen, then lysed using ice-cold radioimmunoprecipitation assay buffer (150 mM NaCl, 1% Triton X-100, 0.5% sodium deoxycholate, 1% SDS, 50 mM Tris-HCl, pH 8.0) supplemented with protease inhibitor mixture (Roche, Espoo, Finland). Thirty *μ*g of the total protein of each sample was separated by 12% sodium dodecyl sulfate polyacrylamide gel electrophoresis (SDS-PAGE) and transferred to a nitrocellulose membrane (Amersham Biosciences, USA). The blocked membranes were then incubated with the CHOP antibody and the immunoreactive bands were visualized using a chemiluminescent reagent as recommended by the Supersignal West Dura Extended Duration Substrate kit (Pierce Chemical, USA). The signals of the bands were quantified using the GS-710 calibrated imaging densitometer (Bio-Rad Laboratories, USA). The results were expressed as a relative density. Equal amount of protein in each lane was confirmed by hybridization with a 1 : 1000 dilution of *β*-actin antibody (Santa Cruz Biotechnology, USA).

### 2.7. Statistical Analysis

The sections were examined at 400 magnifications under the Nikon E200 microscope (Nikon Corporation, Japan) with UTHSCSA Image Tools 3.0 (University of Texas Medical School at San Antonio, TX, USA). The number and optical density of the positive cells were determined. Values were expressed as mean ± SD. The significance of the difference was calculated by a two-tailed Student's *t*-test. *P* < 0.05 was considered statistically significant. 

## 3. Results

### 3.1. The Weight, Blood Glucose, and Urine Glucose Levels in Both Groups

There are no significant differences in weight, blood glucose, and urine glucose levels between two groups before STZ injection (*P* > 0.05). The weight was decreased; blood glucose and urine glucose levels were increased in the diabetic rats compared with those of normal rats six weeks after diabetes induction (*P* < 0.05, Table 1).

### 3.2. Capacity for Learning and Memory

During the 4-day acquisition phase of the Morris water maze test, the escape latency of all rats in the platform quadrant improved gradually, and the escape latency of diabetic rats was obviously longer than that of the control rats (*P* < 0.05, Table 2). Consistent with the escape latency result, the average frequency in finding the platform in 120 s in normal rats was markedly higher than that of the diabetic rats on the 4th day. The results of the Morris water maze test demonstrated that longer escape latency and less frequency in finding the platform in the diabetic rats indicated that diabetes affected the capacity of learning and memory. 

### 3.3. The Pathological Changes and TUNEL Assay of Hippocampal Neurons

**Figure 1:**
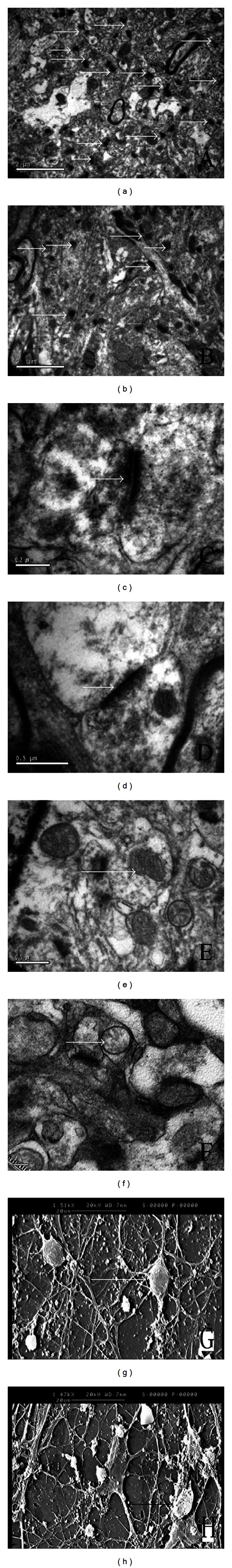
Ultrastructural changes of hippocampus and neurons by TEM and SEM observations: (a, c, e) were control rats, (b, d, f) were diabetes rats, and (g) normal glucose medium (h) 24 h after high glucose treatment. The number of synapses in (b) as the arrows showed decreased compared with (a). The thickness of synaptic density was remarkably decreased, and synaptic cleft was widened in the diabetic rat as arrow of (d) showed. The arrow in (f) showed the mitochondrial crista disappeared and assumed to be characteristics of both apoptosis. The arrow of (h) indexed that membrane was disrupted and reduced neurite in high glucose medium.

**Figure 2:**
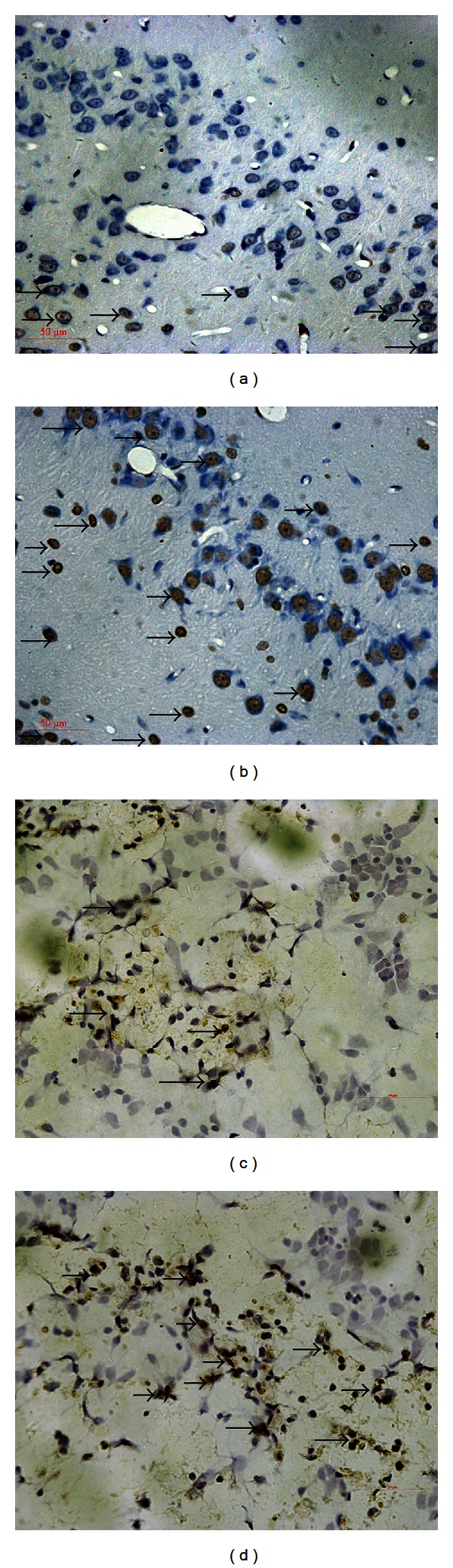
TUNEL-positive neurons of hippocampus in both groups (400x): (a) a control rat, (b) a diabetic rat, (c) control neurons, and (d) 24 h after high glucose treatment neurons. TUNEL positive staining neurons were labeled as brown, the arrows in (b) (diabetic rat) and (d) (neurons of high glucose medium) showed that the number of TUNEL-positive cells was increased when compared with the normal rats (a) and neurons of normal medium (c).

We examined 5 fields in each hippocampus and analyzed all data of synapses in each group. The number of synapses was 0.95 ± 0.23/um^2^ in the dentate gyrus of diabetic rats and 2.0 ± 0.32/um^2^ in the normal rats (*P* < 0.05, Figures 1(a) and 1(b)). The thickness of synaptic density was remarkably decreased while synaptic cleft was widened in the diabetic rat compared with the control rat (16.9 ± 3.8 nm versus 30.1 ± 4.1 nm, 27.1 ± 2.6 nm versus 17.9 ± 2.6 nm, Figures 1(c) and 1(d)). The mitochondrial crista of some neurons disappeared and the vacuoles in the swollen mitochondrial matrix increased, which sometimes assumed to be characteristics of both apoptosis and neurodegeneration (Figures 1(e) and 1(f)). After 24 h culturing in high glucose medium, the notable changes also occurred in neurons which showed distinct degrees of damage as the membrane was disrupted and reduced neurite (Figures 1(g) and 1(h)). The hyperglycemia-induced apoptotic death was determined by the number of TUNEL-positive cells. We examined 5 vision fields in each hippocampus and counted all the cells and apoptotic cell numbers in these fields. The percentage of apoptotic cells in each group was analyzed. The results demonstrated that the percentage of apoptotic cells was increased to 33.1 ± 6.7% in the diabetic rats when compared with the normal rats (4.8 ± 1.7%). The same trend was found in the cultured neurons (54.2 ± 12.6% in high glucose medium versus 9.5 ± 3.2% in normal medium, Figure 2).

### 3.4. The Expression of CHOP in the Hippocampus and Cultured Neurons

**Figure 3:**
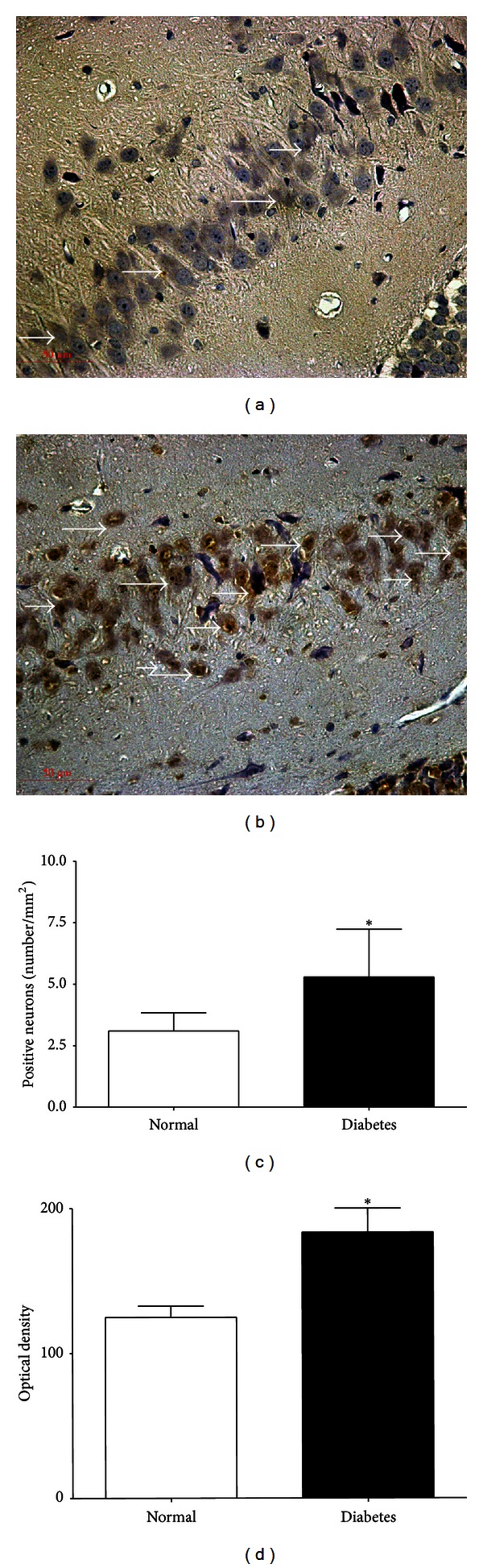
CHOP positive neurons in dentate gyrus of the hippocampus. (400x): (a) a control rat, (b) a diabetic rat, (c) the number of CHOP positive neurons and (d) the optical density of CHOP positive neurons. The immunoreactivity of CHOP protein was visualized in brown granular immunostain pattern and the brown stain indicates increased CHOP activity in diabetic rats (b) compared with the control rats (a). *P < 0.05  versus control rats.

**Figure 4:**
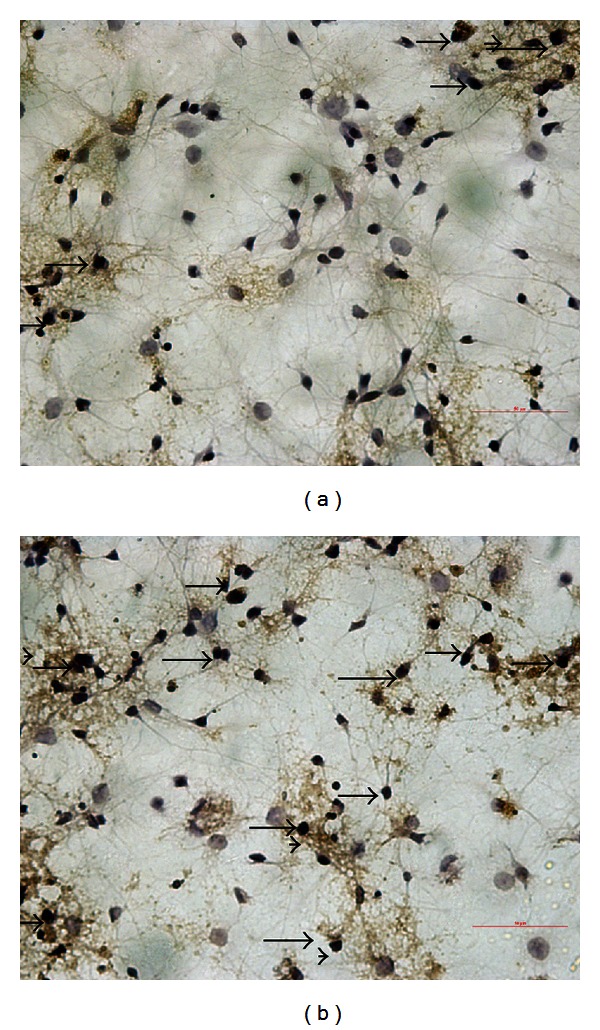
CHOP expression in neurons derived from primary cultured embryonic hippocampal neurons of E18 rats (400×): (a) neurons of the control group and (b) neurons under high glucose treatment; the arrows showed that the neurons of high glucose medium group (b) had numerous fragmentations and degenerated cells with shrunk neuritis and dendrite decreased while the expression of CHOP in neurons under high glucose medium (b) was apparently increased compared with the normal glucose medium (a).

**Figure 5:**
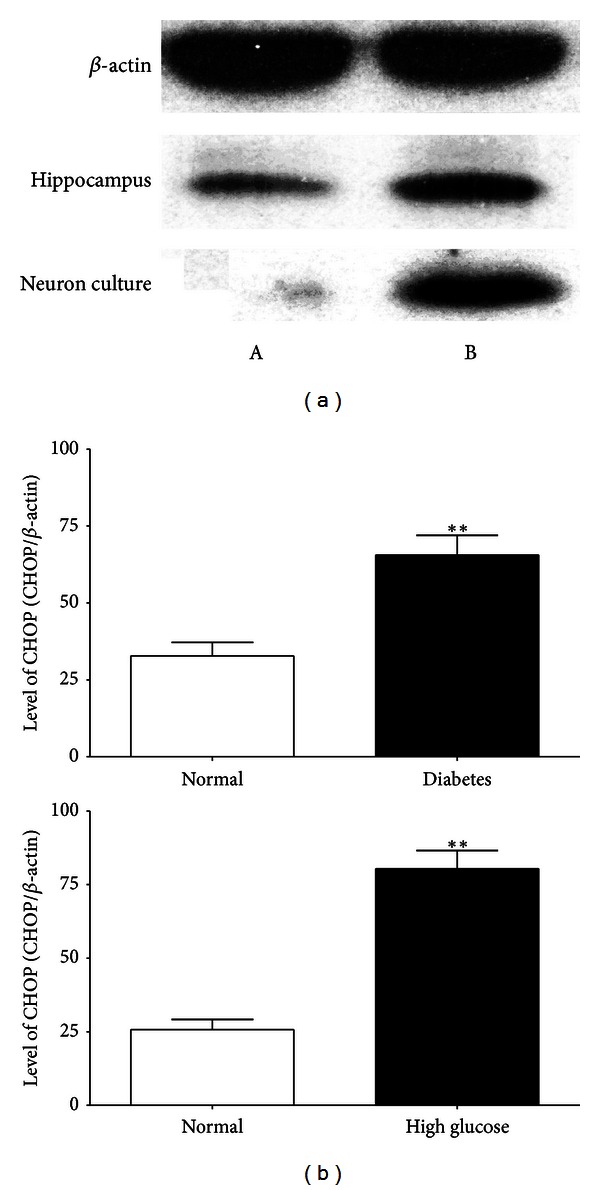
Western blot assays of CHOP in rats' hippocampus and neurons in both groups: (a) control group and (b) diabetes group; **P < 0.05 compared with the control.

The immunoreactivity of CHOP protein was visualized in brown granular immunostain pattern. It was expressed predominantly in the nucleus. Compared to the control group, CHOP positive neurons were significantly increased in the hippocampus of the diabetes group (Figures 3(a) and 3(b)). Quantitative analysis of the number and optical density of CHOP positive neurons in hippocampal area was increased compared with the control rats (*P* < 0.05, Figures 3(c) and 3(d)). From cultured hippocampal neurons, there were obvious morphological differences between the high glucose group and the control group. The neurons of the high glucose medium group had numerous fragmentations and degenerated cells with shrunk neuritis were observed (Figures 4(a) and 4(b)) while the expression of CHOP was apparently increased compared with neurons of normal glucose medium as arrows showed in Figures 4(a) and 4(b). The Western blot assay results demonstrated that the levels of CHOP proteins in the hippocampus of diabetic rats and hippocampal neurons of high glucose medium were markedly augmented when compared with the control group (*P* < 0.05, Figure 5).

## 4. Discussion 

DM patients show an increase in the risk of dementia, may which affect the synapses in hippocampus and induced cognitive and memory impairments [16]. The present study used the Morris water maze test to explore the cognitive function of STZ-induced diabetic rats and found that the escape latency of the diabetic rats was obviously longer and the times of finding the platform in 120 s were less than the control rats. We also found that hyperglycemia could interrupt synaptic structure and induce neuronal apoptosis while the TUNEL positive staining cells increased in diabetic rats and high glucose medium cultured neurons. Previous studies demonstrated that there was a connection between hippocampal synaptic plasticity and memory [28–30]. Our results showed the ultrastructural changes of the neurons and the number of hippocampal synapses decreased, which further led to the impairment of neural connectivity under hyperglycemia. It finally led to the functional and morphological injury of hippocampal synaptic plasticity *in vivo* and *in vitro*. The present results demonstrated that diabetes could reduce the rats' ability of learning and memory, destroyed the structures of the hippocampal neurons and synapses, and induced neuronal apoptosis.

CHOP played a critical role in ER stress-induced apoptosis, and it was implicated in mediating neurodegeneration in Alzheimer's disease (AD) [31] and forebrain ischemia gerbils [32]. The CHOP activation has been observed in neurons undergoing apoptosis due to perturbations in ER calcium levels. Evidence suggests that ER stress is a key link to obesity, insulin resistance, and type 2 diabetes [33], and glucose-induced endoplasmic reticulum stress is independent of oxidative stress [34]. Many neurodegenerative diseases were also tightly associated with ER stress [35]. CHOP mRNA is induced in hippocampus of rats which were subjected to global cerebral ischemia and the underlying reason is the depletion of calcium stores from the ER [36]. The present results implicated a crucial role for CHOP in hyperglycemia-induced apoptosis of hippocampal neurons* in vivo* and *in vitro*. Exposure to high glucose could induce ER stress by the generation of free radicals, aberrant protein glycosylation, or increased membrane and protein turnover. In patients with hyperglycemia, elevated glucose levels might generate reactive oxygen species, require a marked increase in the synthesis of proteins in the neurons, and alter the brain glucose homeostasis [37]. If this was not counterbalanced, the ER would be overwhelmed and initiate apoptosis as the ultimate ER stress. The present study provided a comprehensive picture of the activation of CHOP-mediated apoptosis pathways under diabetes condition involved in neuronal apoptosis and cognition impairment. 

In conclusion, we demonstrated for the first time that hyperglycemia triggered ER stress-mediated apoptosis in hippocampus. The diabetic rats suffered from greater cell apoptosis in hippocampus and worse memory-related performance tests when compared with the normal rats. These findings suggest that CHOP may play an important role in the cognitive impairment of diabetes. However, the detailed mechanisms for the initial ER stress in hippocampus under hyperglycemia need to be clarified in the future.

## Figures and Tables

**Table 1:** The weight, blood, and urine glucose in both groups before and after diabetes induction (*n* = 15).

	Groups	Weight (g)	Plasma glucose (mmol/L)	Urine glucose
Before diabetes induction	Control group	165.3 ± 6.3	4.7 ± 0.6	(−)
	DM group	168.2 ± 7.9^#^	4.6 ± 0.5^#^	(−)^#^
Six weeks after diabetes induction	Control group	383.5 ± 21.7	6.1 ± 1.9	(−)
	DM group	218.9 ± 46.4*	21.8 ± 6.1*	(+++)*

^#^ *P* > 0.05 compared with the control; *P < 0.05 compared with the control.

**Table 2:** The escape latencies and the average frequency in finding the platform in both groups (*n* = 15).

Groups	The escape latency (seconds)			The average frequency in finding the platform in 120 s on 4th day (times)
	Day 1	Day 2	Day 3	
Control group	40.8 ± 27.2	25.5 ± 13.1	12.5 ± 4.3	5.9 ± 3.8
DM group	72.8 ± 34.5*	42.3 ± 12.6*	28.2 ± 18.3*	2.6 ± 1.1*

*P < 0.05 compared with the control.

## Abbreviations

CHOP: C/EBP Homology Protein
DM: Diabetes mellitus
ER: Endoplasmic reticulum
STZ: Streptozotocin
PBS: Phosphate-buffered saline
SEM: Scanning electron microscopy
SDS-PAGE: Sodium dodecyl sulfate polyacrylamide gel electrophoresis
TEM: Transmission electron microscope.

## References

[1] Gray SP, Cooper ME (2011). Diabetic nephropathy in 2010: alleviating the burden of diabetic nephropathy. *Nature Reviews Nephrology*.

[2] Won JC, Kwon HS, Kim CH (2012). Prevalence and clinical characteristics of diabetic peripheral neuropathy in hospital patients with Type 2 diabetes in Korea. *Diabetic Medicine*.

[3] Liu HF, Zhang HJ, Hu QX (2012). Altered polarization, morphology, and impaired innate immunity germane to resident peritoneal macrophages in mice with long-term type 2 diabetes. *Journal of Biomedicine and Biotechnology*.

[4] Negre-Salvayre A, Salvayre R, Augé N, Pamplona R, Portero-Otín M (2009). Hyperglycemia and glycation in diabetic complications. *Antioxidants and Redox Signaling*.

[5] Das Evcimen N, King GL (2007). The role of protein kinase C activation and the vascular complications of diabetes. *Pharmacological Research*.

[6] Haidara MA, Yassin HZ, Rateb M, Ammar H, Zorkani MA (2006). Role of oxidative stress in development of cardiovascular complications in diabetes mellitus. *Current Vascular Pharmacology*.

[7] Ceriello A (2006). Oxidative stress and diabetes-associated complications. *Endocrine Practice*.

[8] Rains JL, Jain SK (2011). Oxidative stress, insulin signaling, and diabetes. *Free Radical Biology and Medicine*.

[9] Liao MT, Sung CC, Hung KC, Wu CC, Lo L, Lu KC (2012). Insulin resistance in patients with chronic kidney disease. *Journal of Biomedicine and Biotechnology*.

[10] XiaoMing Z, Xi Z, Fang S, Jilin Z (2004). Specific changes of somatostatin mRNA expression in the frontal cortex and hippocampus of diabetic rats. *Journal of Anatomy*.

[11] Velayudhan L, Poppe M, Archer N, Proitsi P, Brown RG, Lovestone S (2010). Risk of developing dementia in people with diabetes and mild cognitive impairment. *British Journal of Psychiatry*.

[12] Murthy SB, Jawaid A, Schulz PE (2008). Diabetes mellitus and dementia: advocating an annual cognitive screening in patients with diabetes mellitus. *Journal of the American Geriatrics Society*.

[13] Munshi M, Grande L, Hayes M (2006). Cognitive dysfunction is associated with poor diabetes control in older adults. *Diabetes Care*.

[14] Ahtiluoto S, Polvikoski T, Peltonen M (2010). Diabetes, Alzheimer disease, and vascular dementia: a population-based neuropathologic study. *Neurology*.

[15] Zhang T, Pan BS, Sun GC, Sun X, Sun FY (2010). Diabetes synergistically exacerbates poststroke dementia and tau abnormality in brain. *Neurochemistry International*.

[16] Gaspar JM, Baptista FI, Galvão J, Castilho AF, Cunha RA, Ambrósio AF (2010). Diabetes differentially affects the content of exocytotic proteins in hippocampal and retinal nerve terminals. *Neuroscience*.

[17] Zhou J, Wang L, Ling S, Zhang X (2007). Expression changes of growth-associated protein-43 (GAP-43) and mitogen-activated protein kinase phosphatase-1 (MKP-1) and in hippocampus of streptozotocin-induced diabetic cognitive impairment rats. *Experimental Neurology*.

[18] Madeo F, Kroemer G (2009). Intricate Links between ER Stress and Apoptosis. *Molecular Cell*.

[19] Xiang J, Gu X, Qian S, Chen Z (2010). Endoplasmic reticulum stress-mediated apoptosis involved in indirect recognition pathway blockade induces long-term heart allograft survival. *Journal of Biomedicine and Biotechnology*.

[20] Sheikh-Ali M, Sultan S, Alamir AR, Haas MJ, Mooradian AD (2010). Hyperglycemia-induced endoplasmic reticulum stress in endothelial cells. *Nutrition*.

[21] Trusina A, Papa FR, Tang C (2008). Rationalizing translation attenuation in the network architecture of the unfolded protein response. *Proceedings of the National Academy of Sciences of the United States of America*.

[22] Tagawa Y, Hiramatsu N, Kasai A (2008). Induction of apoptosis by cigarette smoke via ROS-dependent endoplasmic reticulum stress and CCAAT/enhancer-binding protein-homologous protein (CHOP). *Free Radical Biology and Medicine*.

[23] Xu L, Han F, Mandal A, Rao GN, Zhang X (2010). Diazoxide attenuates hypothermic preservation-induced renal injury via down-regulation of CHOP and caspase-12. *Nephrology Dialysis Transplantation*.

[24] Nakka VP, Gusain A, Raghubir R (2010). Endoplasmic reticulum stress plays critical role in brain damage after cerebral ischemia/reperfusion in rats. *Neurotoxicity Research*.

[25] Galehdar Z, Swan P, Fuerth B, Callaghan SM, Park DS, Cregan SP (2010). Neuronal apoptosis induced by endoplasmic reticulum stress is regulated by ATF4-CHOP-mediated induction of the Bcl-2 homology 3-only member PUMA. *Journal of Neuroscience*.

[26] Salminen A, Kauppinen A, Suuronen T, Kaarniranta K, Ojala J (2009). ER stress in Alzheimer’s disease: a novel neuronal trigger for inflammation and Alzheimer’s pathology. *Journal of Neuroinflammation*.

[27] Fonseca SG, Burcin M, Gromada J, Urano F (2009). Endoplasmic reticulum stress in *β*-cells and development of diabetes. *Current Opinion in Pharmacology*.

[28] Bruel-Jungerman E, Rampon C, Laroche S (2007). Adult hippocampal neurogenesis, synaptic plasticity and memory: facts and hypotheses. *Reviews in the Neurosciences*.

[29] Kotilinek LA, Westerman MA, Wang Q (2008). Cyclooxygenase-2 inhibition improves amyloid-*β*-mediated suppression of memory and synaptic plasticity. *Brain*.

[30] Arendt T (2009). Synaptic degeneration in Alzheimer’s disease. *Acta Neuropathologica*.

[31] Hoozemans JJM, Veerhuis R, Van Haastert ES (2005). The unfolded protein response is activated in Alzheimer’s disease. *Acta Neuropathologica*.

[32] Oida Y, Shimazawa M, Imaizumi K, Hara H (2008). Involvement of endoplasmic reticulum stress in the neuronal death induced by transient forebrain ischemia in gerbil. *Neuroscience*.

[33] Ozcan U, Yilmaz E, Özcan L (2006). Chemical chaperones reduce ER stress and restore glucose homeostasis in a mouse model of type 2 diabetes. *Science*.

[34] Mooradian AD, Haas MJ (2011). Glucose-induced endoplasmic reticulum stress is independent of oxidative stress: a mechanistic explanation for the failure of antioxidant therapy in diabetes. *Free Radical Biology and Medicine*.

[35] Ito D, Suzuki N (2009). Seipinopathy: a novel endoplasmic reticulum stress-associated disease. *Brain*.

[36] Yamauchi T, Sakurai M, Abe K, Matsumiya G, Sawa Y (2007). Impact of the endoplasmic reticulum stress response in spinal cord after transient ischemia. *Brain Research*.

[37] da-Silva WS, Gómez-Puyou A, de Gómez-Puyou MT (2004). Mitochondrial bound hexokinase activity as a preventive antioxidant defense. Steady-state ADP formation as a regulatory mechanism of membrane potential and reactive oxygen species generation in mitochondria. *Journal of Biological Chemistry*.

